# Seasonal biogeochemical cycling of permeable sediments in a temperate shelf sea environment

**DOI:** 10.1007/s10533-026-01365-5

**Published:** 2026-08-15

**Authors:** S. E. Reynolds, C. E. L. Thompson, W. B. Homoky, M. Williams, J. Klar, L. O. Amoudry, V. Kitidis, B. Silburn, L. Chapman-Greig, R. E. Parker, E. M. S. Woodward, P. J. Statham, G. R. Fones

**Affiliations:** 1 School of the Environment & Life Sciences, University of Portsmouth, King Henry Building, King Henry I Street, Portsmouth, PO1 2DY UK; 2 School of Ocean and Earth Science, University of Southampton, National Oceanography Centre, European Way, Southampton, SO14 3ZH UK; 3 School of Earth and Environment, University of Leeds, Leeds, LS2 9JT UK; 4 Departamento de Ingeniería Hidráulica y Ambiental, Pontificia Universidad Católica de Chile, 7820436 Santiago, Chile; 5 National Oceanography Centre, Joseph Proudman Building, 6 Brownlow Street, Liverpool, L3 5DA UK; 6 Plymouth Marine Laboratory, Prospect Place, Plymouth, PL1 3DH UK; 7 Cefas, Pakefield Road, Lowestoft, Suffolk NR33 0HT UK

**Keywords:** Celtic Shelf Sea, Permeable sediment, Oxygen consumption, Shelf Sea Biogeochemistry

## Abstract

In highly productive shelf-seas, permeable sediments cover the majority of the benthic environment. However, most research has focussed on shallow environments where currents, tidal flows, terrestrial inputs and benthic productivity have a greater influence than in sediments in deeper shelf areas. Given the extent of permeable sediments across shelf-seas globally, it is imperative to ascertain their biogeochemical function. Here, we present for the first time a seasonal study of biogeochemical processes in deep temperate shelf-sea permeable sediments. Celtic Sea (North-Eastern European shelf) sediments were incubated to establish rates of consumption or production of oxygen (O_2_), nutrients and iron. Incubations remained oxic throughout, with a significant difference in O_2_ consumption across seasons, with rates post-bloom (-15.65 ± 0.99 μmol O_2_ L^−1^ h^−1^) two-fold higher than pre-bloom (-9.60 ± 1.68 μmol O_2_ L^−1^ h^−1^) and late-summer (-9.05 ± 0.68 μmol O_2_ L^−1^ h^−1^). There was a consistent release of phosphate and silicate across seasons and drawdown of nitrate post-bloom. A significant production of iron(II) was observed post-bloom. We find that the seasonal differences observed are driven by changes in the delivery of organic carbon from the pelagic environment and the advective flow through the sediment. Areal estimations of O_2_ fluxes across permeable sediments of the Celtic Shelf Sea reveal ~ 3 × 10^4^ mol O_2_ d^−1^ are consumed equating to ~ 10 kmol C yr^−1^ being remineralised. Our findings provide direct evidence that advective flow in deep permeable sediments plays a key role in the biogeochemical cycling of nutrients and carbon in shelf seas globally.

**Supplementary Information:**

The online version contains supplementary material available at https://doi.org/10.1007/s10533-026-01365-5.

## Introduction

Continental shelf seas cover 6–8% of the total ocean (Sverdrup et al. 1942), yet with an annual primary production of ~ 11 Gt C yr^−1^ (Jahnke 2010) these regions are responsible for ~ 22% of global primary production (Field et al. 1998). With up to 50% of this fixed carbon reaching the sediment–water interface (Stahl et al. 2004), shelf sediments play a key role in the global biogeochemical cycling of carbon and nutrients (Jahnke et al. 2005).

Relict sands cover approximately 65–70% of continental shelves (Emery 1968; Huettel and Rusch 2000; Janssen et al 2005) and given their high permeability (> 10^−12^ m^2^; Huettel & Rusch 2000; Huettel et al. 1996) advective transport dominates interstitial biogeochemical processes. In shallow shelf seas in particular (< 100 m in water depth), bottom water flushing of sediments is enhanced by significant wave effects (Huettel and Rusch 2000; Precht and Huettel 2003), which control the dynamics of sediment suspension, deposition and transport over large areas of the shelf (Precht and Huettel 2003). Advective transport through permeable sediments and across the sediment water interface is driven by pressure differentials, deriving from the interaction between wave and current-driven flows, local topography and roughness elements (Thibodeaux and Boyle 1987; Huettel and Rusch 2000; Precht and Huettel 2003; Precht et al 2004; Janssen et al 2005), and to a lesser extent, from shear (Huettel and Rusch 2000), wave pumping (Huettel and Rusch 2000; Precht and Huettel 2003; Janssen et al 2005), and biological influences (Volkenborn et al 2007). Advective flow can penetrate tens of centimetres into the seabed (Volkenborn et al 2007) with estimations that globally continental margins filter ~ 33 L m^−2^ d^−1^ of seawater (Riedl et al 1972).

Solute transport associated with advective flow through shelf sediments can be several orders of magnitude larger than that driven by molecular diffusion (Huettel and Webster, 2000; Precht and Huettel 2003; Janssen et al 2005). Consequently, advective flow can heavily impact the supply of oxygen in surface sediments, in particular the depth of oxygen penetration, and the coupled cycling of carbon and nutrients. Since permeable shelf sediments are characterised by low concentrations of organic matter content, they were once considered trivial players in the global biogeochemical cycling of carbon and nutrients (Boudreau et al. 2001). Subsequent advances in the measurement of biogeochemical processes within permeable sediments, however, have demonstrated that these sandy systems are highly active in the cycling of organic matter (Huettel et al. 2014).

Permeable sediment systems are able to entrain organic matter without net accumulation, and the advective supply of oxygen to the pore water supports high rates of organic matter remineralisation and nutrient release (Huettel and Rusch 2000). By contrast, and in the absence of appreciable biological-irrigation, oxidation rates of organic matter in finer grained cohesive sediments are rate-limited by the comparatively slow supply of oxygen from the water column by molecular diffusion. Advection enables permeable sediments to sustain oxygen consumption rates (an indicator of respiration and organic matter remineralisation rates) comparable to organic-rich cohesive sediments (Huettel et al. 2014). Due to hydrodynamic variations above the seabed, the supply and subsurface transport of oxygen through coarse grained permeable sediments is also variable. Under conditions of enhanced advection driven by the processes as discussed above, the rate of oxygen consumption increases demonstrating the role of advected oxygen supply in the benthic remineralisation of organic matter (Janssen et al. 2005). This filtration and recycling function of permeable sediments provides a direct and significant link between the benthic and pelagic environment, with the interactions potentially influencing the biogeochemical processes of the shelf seas (Huettel et al. 2014). Furthermore, advection through permeable sediments, can enhance benthic-pelagic coupling by several of orders of magnitude when compared with the diffusive fluxes from cohesive sediments (Ahmerkamp et al. 2017).

Given the variable supply of O_2_ to permeable sediments and the resultant changes in the sediment redox state, the biogeochemistry of the electron acceptors e.g. nitrate (NO_3_^−^) and iron (Fe), at play should also be considered (Zhou et al. 2023). In an oxic state, NO_3_^−^ from nitrification could potentially be released into pore waters whilst Fe would remain in the solid phase. Alternatively, during a state of anoxia, NO_3_^−^ would be lost from the system via denitrification and Fe would undergo reductive dissolution to produce Fe(II). Additionally, the reductive dissolution of Fe(III) oxides can also result in releases of phosphorus to sediment pore waters (Slomp et al. 1998). Given the role that these elements play in the productivity of the ocean it is imperative to ascertain if temperate shelf permeable sands are a source or sink for these key nutrients.

The majority of the research conducted on the biogeochemistry of permeable sediments has been focussed on the intertidal zone, near-shore or shallow shelf (< 40 m). In these regions, benthic primary production plays a significant role in biogeochemical cycling. In addition, terrestrial inputs from both surface and ground waters (Burnett et al. 2003; Santos et al. 2021) are important and lead to higher nutrient and carbon loads which in-turn enhance primary production in the system. Bed sediments are also constantly being shifted and reworked under the combined actions of currents and waves (Huettel et al. 2014). However, the mean depth of the global shelf regions lies at 160 m (median of 258 m) (Paris et al 2016), where light cannot penetrate to such depths and terrestrial inputs are less significant. Even though sediments at these depths can still be influenced by tides and waves, sediment reworking is episodic as evidenced by observations of static ripples (Thompson et al. 2017) and resuspension of permeable sediments is minimal (Thompson et al. 2019; Williams et al., 2019). Furthermore, benthic biogeochemical cycles are strongly influenced by the seasonality of primary production and organic sedimentation that are coupled to light, temperature and nutrient dynamics in the upper ocean, all of which differ greatly across the gradients from coastal to shelf seas. Thus, there is a gap in our knowledge and understanding of permeable sediment biogeochemistry across seasonal conditions from a site representative of a temperate shelf sea. To address this, we performed a series of microcosm investigations of seasonal biogeochemical cycling in a permeable sediment from the Celtic Sea.

**Fig. 1:**
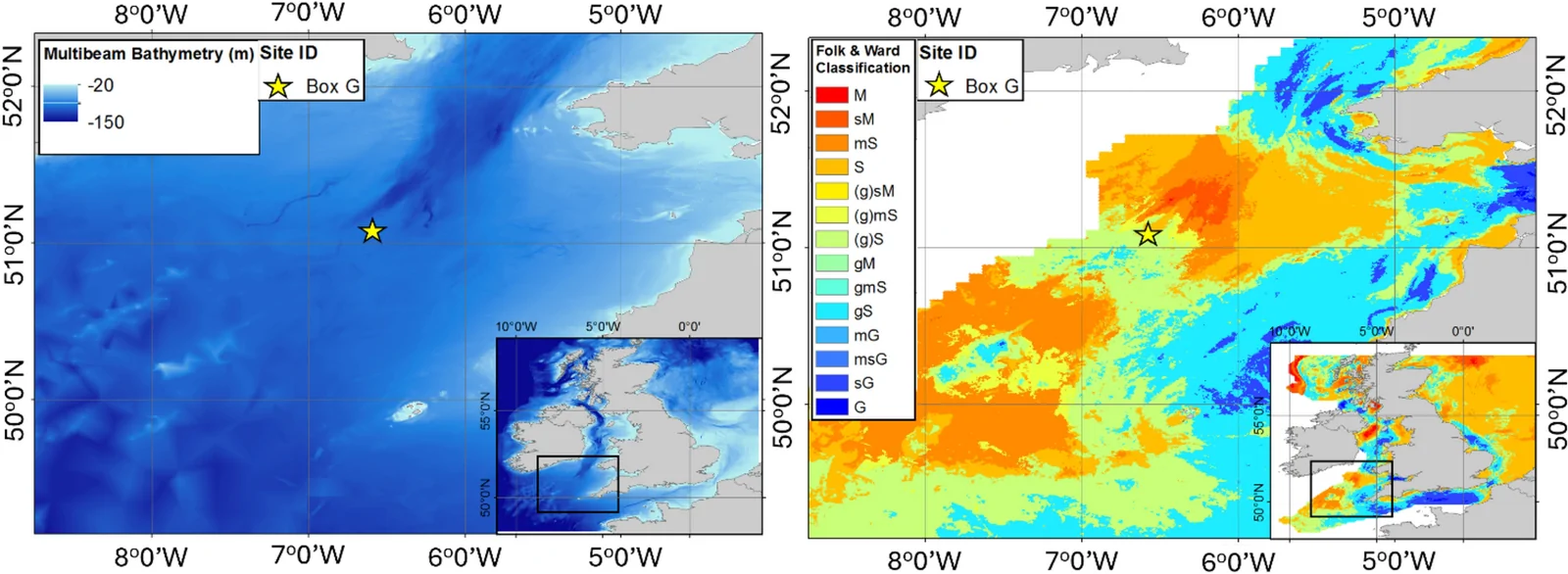
Adapted from Thompson et al. (2017) a map of spatial variations in (**A**) bathymetry, relative to Chart Datum based on 6 arcsec Defra Digital Elevation Map (Astrium, 2015); and **B** surface sediment type using simplified Folk textural classifications, based on BGS surface sediment maps (Stephens 2015; Stephens and Diesing 2015; Folk 1954; Folk and Ward 1957) for the study area. The permeable sampling site is indicated with a star

The temperate Celtic Sea lies between the southwest United Kingdom and North Atlantic Ocean. It covers an area of approximately 70,000 km^2^ on the northwest European continental shelf, lying between depths of 50–250 m (Fig. 1a). This seasonally stratified shelf sea is a net sink for atmospheric carbon dioxide (Kitidis et al., 2017) partially due to the accumulation of a long-lived carbon-rich organic matter pool (Humphreys et al., 2019) and exhibits a strong benthic-pelagic coupling (Kitidis et al. 2017). Sediments in the Celtic Sea comprise a gradient of lithologies from fine muds to gravels (Stephens and Diesing 2015). Pure sandy sediments account for 16% of the Celtic Sea area, with other permeable sediments (including gravelly sands) accounting for approximately 64% of the total sediment area (Stephens 2015; Stephens & Diesing 2015; Thompson et al. 2017).

We report findings from three expeditions to the southern Celtic Sea in 2015 as part of the UK-led Shelf Sea Biogeochemistry Programme (Thompson et al. 2017). During these voyages we sampled a permeable sediment located at 100 m depth in pre-bloom (March), post-bloom (May) and late summer (August) conditions. The samples are used to determine the seasonal variation in oxygen consumption rate, and associated fluxes of Fe(II), inorganic nutrients, dissolved organic carbon (DOC) and dissolved organic nitrogen (DON) using a purpose-built flow-through reactor. We find evidence for a strong seasonal variation in biogeochemical cycling linked to the spring bloom, and significant associated fluxes of oxygen, iron(II) and nutrients that call for wider assessment and integration of permeable sediments into all aspects of continental shelf biogeochemistry and ecosystem services.

## Methodology

### Field campaign

Three research voyages on board the RRS Discovery were conducted in the southern Celtic Sea in 2015 and were timed to sample pre-bloom (1st – 26th March), post-bloom (4th – 25th May) and late summer (6th August – 2nd September) conditions. A permeable site was identified (Thompson et al 2017) at locality 51°4.3569 N, 6°34.866 W (Fig. 1) with a water depth of ~ 100 m. All sediment samples were collected using a 0.1 m^2^ NIOZ box corer which was subsequently sampled via sub-cores or other methods (see below for further details).

### Site characterisation and flow at bed

For particle size analysis 10 cm sub-cores were collected and subsequently sliced in 1 cm aliquots over the upper 5 cm. The 1 cm aliquots of sediment were washed over a 63 mm sieve to separate the fine and course material whilst retaining all of the sample. The course fraction was analysed using Sieve analysis (Mason 2011) to half-phi resolution, whilst the fines were analysed on a Coulter LS200 Laser Sizer. The distribution was combined and statistics calculated according to the Folk and Ward (1957) geometric (modified) graphical measured and Folk (1954) textural classes.

For measurements of bulk density, 50 ml syringe sub-cores were collected. Samples were extruded and sliced into 1 cm sections and the sediment was weighed, dried and weighed again. The dry weight was used to calculate both the bulk density and porosity (Burdige, 2021). The specific permeability was calculated according to Engelund (1953).

Determination of the oxygen penetration depth (OPD) was made from 10 cm sub-cores collected with the overlying water retained. Upon collection the sub-core was immediately profiled using a Unisense OXY-500 microsensor at 1 mm measurement intervals. From this the OPD was established as the last depth at which measurable oxygen in the sediment pore waters is detected (Cai & Sayles 1996).

Sediment samples for particulate organic carbon analysis (POC) were collected from the same homogenised sediment used in the flow reactor set up (see below for details). Samples were stored at − 20 °C until further analysis at the University of Portsmouth, UK. Samples were dried at 40 °C, ground and acidified following the protocol of Phillips et al (2011). Analysis was performed in triplicate using a Thermo Flash 2000 CHNS/O analyser.

Samples for porewater nutrients and diffusive nutrient fluxes were collected and analysed as described in Thompson et al (2017).

Images of the sediment in-situ were made using Sediment Profile Imagery (SPI) to acquire vertical profile images of the seabed (Rhoads and Cande 1971). The system comprises of a water filled mirrored prism, which is gravity driven into the seabed as the system is lowered, at which point a camera is triggered to acquire images of the sediment–water interface. The camera set-up consisted of a Nikon D100 digital camera with a 35 mm lens and self-contained strobe flash unit.

The magnitude of porewater flow was estimated based on a combination of in situ measurements and outputs of wave models. The magnitude of porewater flow induced by the interaction between near-bed flow and bed topography was calculated from in situ measurements of near-bed currents and bedform dimensions. In our case, near-bed velocities were measured by an Acoustic Doppler Velocimeter (Nortek Vector) and small-scale bedforms were measured by a three-dimensional Acoustic Ripple Profiler (Thorne and Hanes, 2002; Marine Electronics, 2009). Both instruments were mounted on a benthic tripod and deployed in situ for a few tidal cycles. These short deployments did not enable to capture wave events that would result in wave pumping. Instead, the magnitude of porewater flow generated by wave pumping (i.e. due to the pressure gradient from surface gravity waves) was estimated for the Celtic Sea from a wave climate (Bricheno et al., 2015) and linear wave theory for intermediate water depth.

### Experimental setup

A flow through reactor approach was employed and adapted from Rao et al., (2007) that emulates the advective flow through a permeable sediment. Sediment samples were collected from a NIOZ box corer whereby the top 5 cm of sediment was carefully collected to ensure that only the oxic layer was retained. Multiple box cores taken from the same location were used to ensure ample sediment was collected. The sediment was homogenised, with large shell debris removed, and packed into triplicate acrylic core tubes (internal diameter 8 cm; length 15 cm). Once sealed, inflow and outflow connections were made to the bottom and top of the core tubes respectively. Filters (Whatman GF/C) were placed on the underside of the top lid to prevent loss of sediment. Reactors were wrapped in black polythene to exclude light. The inflow water was bottom seawater from the sample site, collected using a titanium CTD rosette fitted with trace metal clean Niskin bottles prior to sediment sampling. The collected seawater was handled under trace metal clean protocols, filtered through a 0.2 μm capsule filter (Sartorius Sartobran 300, cellulose acetate membrane) stored in 20 L carboys in the dark at 9 °C until required. Filtered seawater was aerated for > 12 h prior to and throughout incubations. Inflow water was pumped through the reactors at a constant flow rate of 1 ml min^−1^ via a peristaltic pump which, provided realistic flow rates (velocity of 1.16 cm hr^−1^) however, it did not capture the episodic nature of porewater exchange (Rao et al., 2007). The incubations were conducted in a temperature-controlled laboratory on board the RRS Discovery and maintained at the sea bottom temperature as determined during sampling (Table 1). Incubations were conducted in triplicate and ran for at least 14 days (time restricted by cruise duration) with collection of samples beginning once a constant outflow was observed, typically 2 h after flow had commenced. Outflow samples were collected twice daily until stability in oxygen concentrations were witnessed (~ 5 days) after which sampling was reduced to once a day.

**Table 1:** Site characteristics for the three seasons during the field campaign

	Pre-bloom	Post-bloom	Late summer
	*March 2015*	*May 2015*	*August 2015*
Bottom temperature (°C)	8	9	11
Sample type	Unimodal, moderately well sorted		
Textural group	Slightly gravelly sand		
Sediment name	Slightly very fine gravelly medium sand		
Median grain size D_50_ (μm)	343.1	307.0	371.6
Bulk density*	1299.84 ± 385.98	1412.68 ± 332.69	1714.14 ± 84.74
Porosity average*	0.51 ± 0.15	0.47 ± 0.13	0.35 ± 0.03
Permeability (m^2^)	8.33E-12	4.92E-12	5.92E-12
Particulate organic carbon %	0.15 ± 0.01	0.30 ± 0.17	0.13 ± 0.01
Oxygen penetration depth (cm)	5	1.4	0.5

^*^ represent average of 1 cm slices of upper 5 cm, ± 1 standard deviation

In addition to the incubations conducted throughout the at-sea field campaign, additional incubations were conducted post-cruise to investigate the effect of the residence time of the flow through reactors on the biogeochemical cycling of a permeable sediment. During the post-bloom cruise in August 2015, permeable sediment (with overlying seawater) and filtered bottom seawater (collected as described previously) was retained (stored in the dark at 4 °C and sediment regularly homogenised to ensure anoxia did not occur) and returned to the National Oceanography Centre, Southampton, UK. To emulate a reduced residence time (Rao et al., 2007) a shorter core tube of 10 cm was used, while to increase the residence time a longer core tube of 32 cm was used, both with an internal diameter of 8 cm. The same packing procedure and experimental set-up was followed as had been conducted whilst at sea. The incubations were conducted in a temperature-controlled laboratory at sea bottom temperature in August 2015 (11 °C). Given the limitations in resources and time, these experiments could not be run in triplicate and were only run once.

### Sampling and analysis

The outflow from the flow through reactors was sampled for a suite of parameters. Sample lines from the outflow were typically between 5–10 cm in length. Oxygen measurements from both the inflow carboy and outflow were made using a Unisense oxygen microelectrode fitted with flow through glass tubing. Measurements were made as per manufacturers protocol with calibrations conducted daily.

Samples for dissolved organic carbon (DOC) and total dissolved nitrogen (TDN) were collected and filtered through pre-combusted (450 °C, > 4 h; 25 mm GF/F Whatman) filters using Swinnex filter houses. All consumables were cleaned in 10% HCl and thoroughly rinsed in ultra-pure water (18.2 MΩ, Merk Millipore) prior to use. Samples were retained into 20 ml acid cleaned and pre-combusted (450 °C, > 4 h) glass vials (clear 22 mL screw top vials; Sigma 27,173). Samples were acidified (60 μL, 50% phosphoric acid) prior to being capped and stored at 4 °C until analysis. Analysis was conducted on a Shimadzu TOC-V_CPN_ by high temperature catalytic oxidation (HTCO). Samples were analysed at least in triplicate with a typical error of < 5% of the measurement. Certified reference materials (Hansell Laboratory, University of Miami) were run daily with precision on average of ± 5% of the certified value. Dissolved organic nitrogen concentrations were calculated by subtracting the inorganic nitrogen concentrations (nitrate, nitrite and ammonium) from the TDN concentrations.

In reference to all sample collection and analysis for dFe(II), sample and reagent bottles were low density polyethylene (LDPE) or polypropylene and cleaned in 2% Decon, followed by 20% HCl for a week. Reagents were trace-metal grade and made up with ultra-pure water. From the outflow, three sub-samples we collected into vials pre-loaded with ferrozine to directly fix any dFe(II) in the sample. Prior to analysis samples were filtered with 0.22 μm, 25 mm diameter Millex-GP with a mixed cellulose esters membrane. Iron(II) was measured on-board on the day of collection, with a modified spectrophotometric ferrozine method (Stookey 1970; Klar et al. 2017). The limit of detection, determined as three times the standard deviation of the blank was 0.7 nM. The typical uncertainty was < 5% of the measurement.

Samples for inorganic nutrient analysis were collected and filtered through pre-combusted (450 °C, > 4 h) 25 mm GF/F (Whatman) filters using Swinnex filter houses, retained in sterilin tubes and stored at 4 °C until analysis. All the nutrient samples collected at sea were analysed by a Bran and Luebbe segmented flow colorimetric nutrient autoanalyser, fitted with high resolution colorimeters. The analytical chemical methodologies used were according to Brewer and Riley (1965) for nitrate, Grasshoff (1976) for nitrite, Mantoura and Woodward (1983) for ammonium, and Kirkwood (1989) for silicate and phosphate. Samples were either analysed without dilution, or where required, diluted with low nutrient seawater. Strict protocols were followed during sample/standard handling and reagent preparation (Hydes et al 2010). Nutrient reference materials (KANSO, Japan) were analysed each day to check precision and accuracy. Precision was between 2 and 3%, with limits of detection of 0.02 μM for nitrate, silicate and phosphate, and 0.01 μM for nitrite and 0.03 μM for ammonium. Nutrient samples collected during the on-shore laboratory-based experiments were filtered as described previously and fixed with mercuric chloride (HgCl_2_) until further analysis. Sample analysis was conducted at Portsmouth University and achieved using a SEAL analytical QuAAtro segmented flow analysis system.

### Data analysis

The advective rates of consumption and production of the solutes were calculated following Ahmerkamp et al. 2017. To determine the velocity (1) of the flow through the sediment-packed columns

1 $$u=\frac{Q}{A}$$

where *u* is the velocity (cm hr^−1^), *Q* is the flow rate (ml min^−1^) and *A* is the cross-sectional area of the column (cm^−2^). The residence time (*rt*) (2) was calculated where *L*_*c*_ is the column length (cm).

2 $$rt=\frac{ {L}_{c}}{u}$$

The advective rates (L hr^−1^) (3) for all solutes were subsequently calculated. *C*_*out*_ is the outflow measurement and *C*_*in*_ the inflow measurement of the solute.

3 $$\frac{{(C}_{out}- {C}_{in})}{rt}$$

ANOVA tests were conducted using SigmaPlot v14.0 (SyStat Software Inc.) to determine the significance of differences between nutrient profiles and advective rates across seasons. Instances where the normality test passed, Tukey’s post hoc analysis was run for each ANOVA test to identify which parameters were responsible for any significant differences found. If the normality test failed, then Dunn’s post hoc analysis was applied.

## Results

### Physical sediment properties

**Fig. 2:**
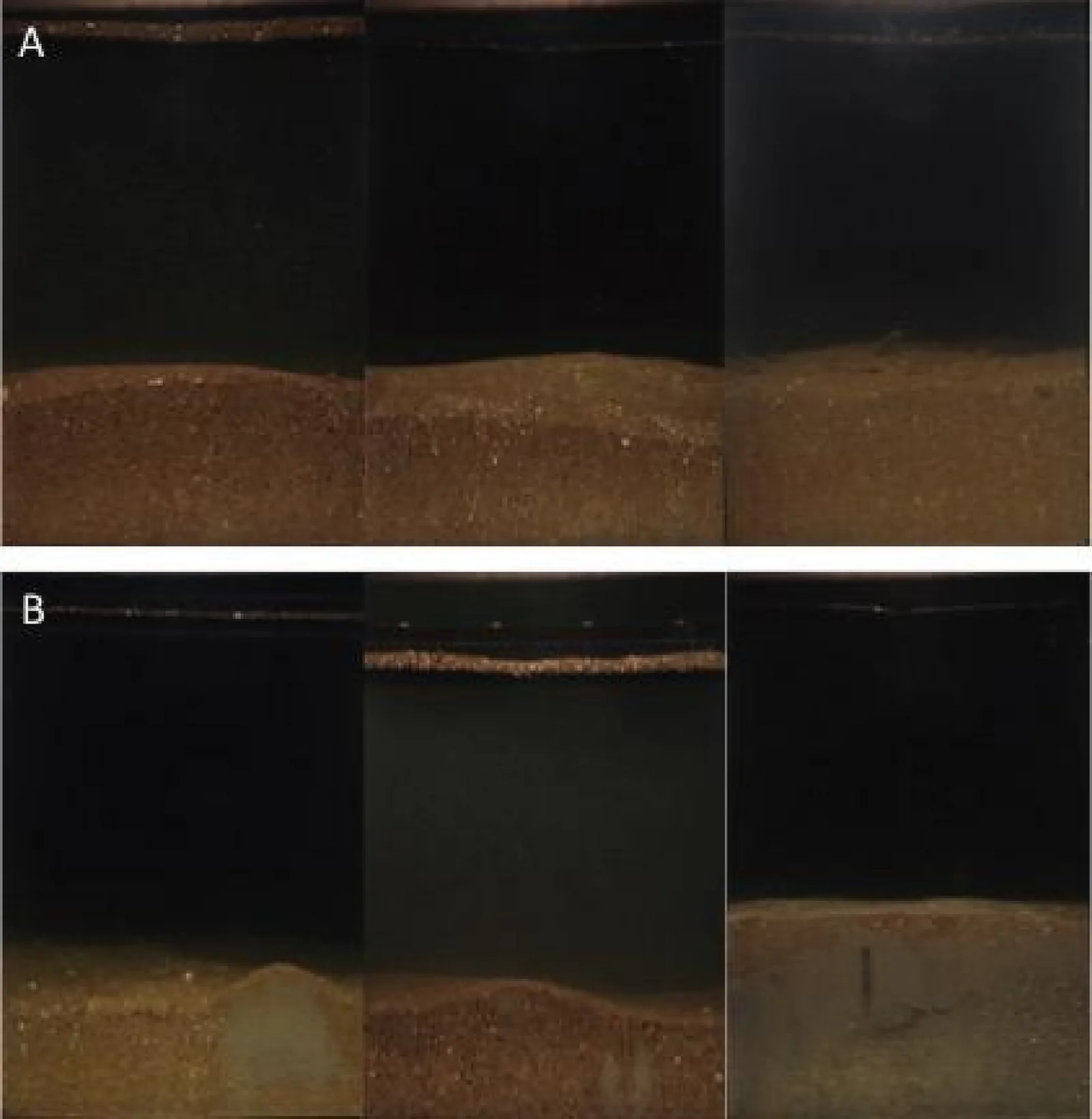
SPI images taken from the permeable site. Images demonstrate the heterogeneity of the site with (**A)** showing a more homogenous characteristic with relatively clean sand, whilst (**B**) provides evidence of the presence of mud lenses within the permeable sediment. Evidence of ripples can be seen in both sets of images

Across all seasons, the sediment from the permeable sampling site was classified as unimodal, moderately well sorted with a textural group classification of a slightly gravelly sand (Table 1). SPI images (Fig. 2) reveal the heterogeneity of the permeable sediment sampling site and coincide with what was observed by ourselves during sampling and processing of the sediment. Lenses of fines were apparent across all seasons, with no obvious seasonal trends. However, sediment classification analysis demonstrates a decrease in the percentage of fine material (Supplementary Table S1) across the seasonal sampling programme. Bulk density of the upper 5 cm of the sediment increases across the seasons from 1299.84 ± 385.98 pre-bloom, to 1412.68 ± 332.69 post-bloom and 1714.14 ± 84.74 late summer. Coinciding with this increase in bulk density, porosity decreased across seasons from 0.51 ± 0.15 pre-bloom to 0.35 ± 0.03 late summer. Permeability was greatest pre-bloom which aligns with the lowest bulk density, highest porosity and deepest OPD. Here, the permeability was calculated from the sediment grain size using the simple permeability model of Engelund (1953) which assumes a uniform sediment size. Whilst the permeability of the sediment reported in this study should perhaps be taken with some caution, studies have demonstrated that model-based assessments do align well with in-situ measurements of permeability (Neumann et al. 2017). Organic carbon was highest post-bloom (0.30 ± 0.17%), indicating the deposition of the pelagic spring bloom to the seafloor, with a halving of organic carbon loading pre-bloom and late summer. Oxygen penetration depth (OPD) decreased across seasons from 5 cm pre-bloom to 0.5 cm late summer.

Near-bed (30 cm above the bed) tidal currents reached speeds of approximately 40 cm s^−1^, and ripples were 1–3 cm high and 15–30 cm long (Thompson et al. 2017). These in situ measurements of bed ripples also showed negligible ripple migration, so trapping/release of porewater by ripple migration can be neglected. The porewater transport velocity is then proportional to the pressure gradient over the ripple, and inversely proportional to the ripple wavelength (e.g., Rutherford et al., 1995; Precht and Huettel, 2003). For ripples under wave-generated near-bed oscillatory flows, we estimated the pressure gradient as a function of the near-bed velocity and ripple dimensions following Shum (1992, 1993), the near-bed orbital wave velocity from linear wave theory and wave climate (Bricheno et al., 2015), and find porewater transport velocities under extreme wave events to reach approximately 2 cm h^−1^. For ripples under tidal currents, the pressure gradient scales with the square of the near-bed tidal velocity, and smaller porewater transport velocities are obtained to be approximately 1–2 cm h^−1^.

### Consumption and production of solutes.

**Fig. 3:**
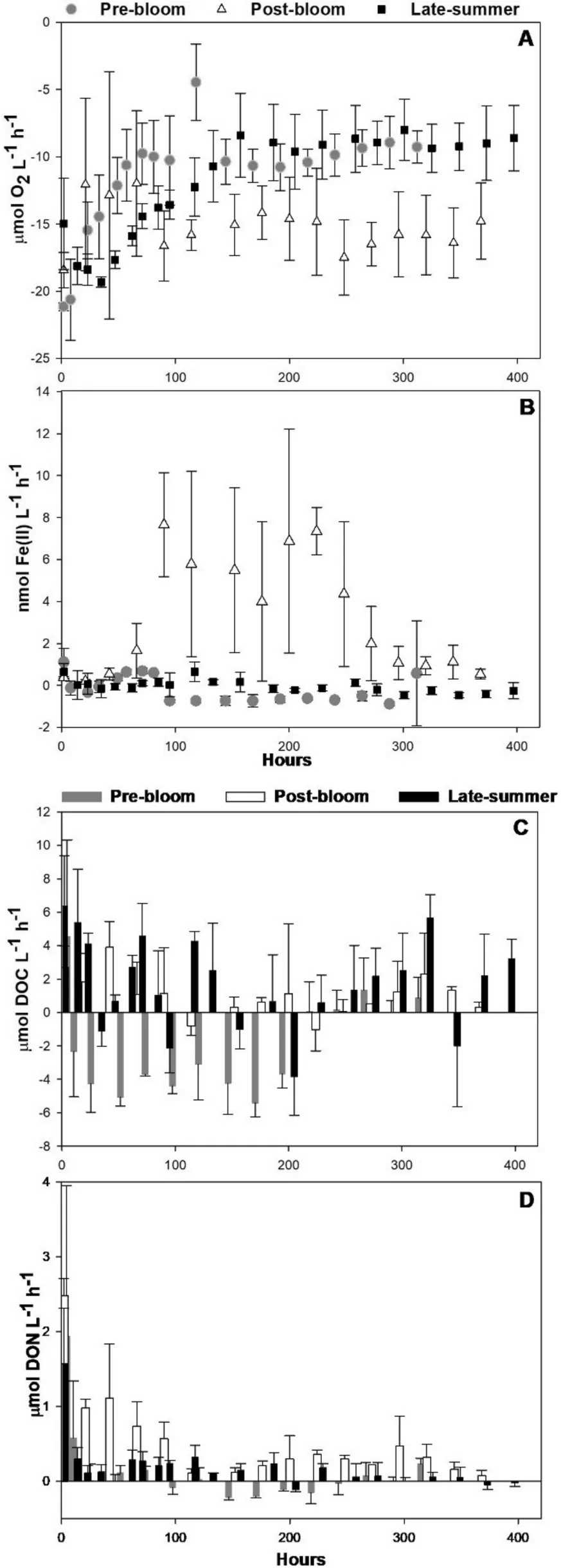
Seasonal rates (pre-bloom grey circles/bars; post-bloom open triangles/bars; late summer black squares/bars) from FTR incubations of **A** O_2_ consumption (μmol O_2_ L^−1^ h^−1^) **B** Fe(II) (nmol L^−1^ h^−1^) (C) DOC (μmol L^−1^ h^−1^) and (D) DON (μmol L^−1^ h^−1^). Error bars represent ± 1 standard deviation of triplicate incubations within each season

**Fig. 4:**
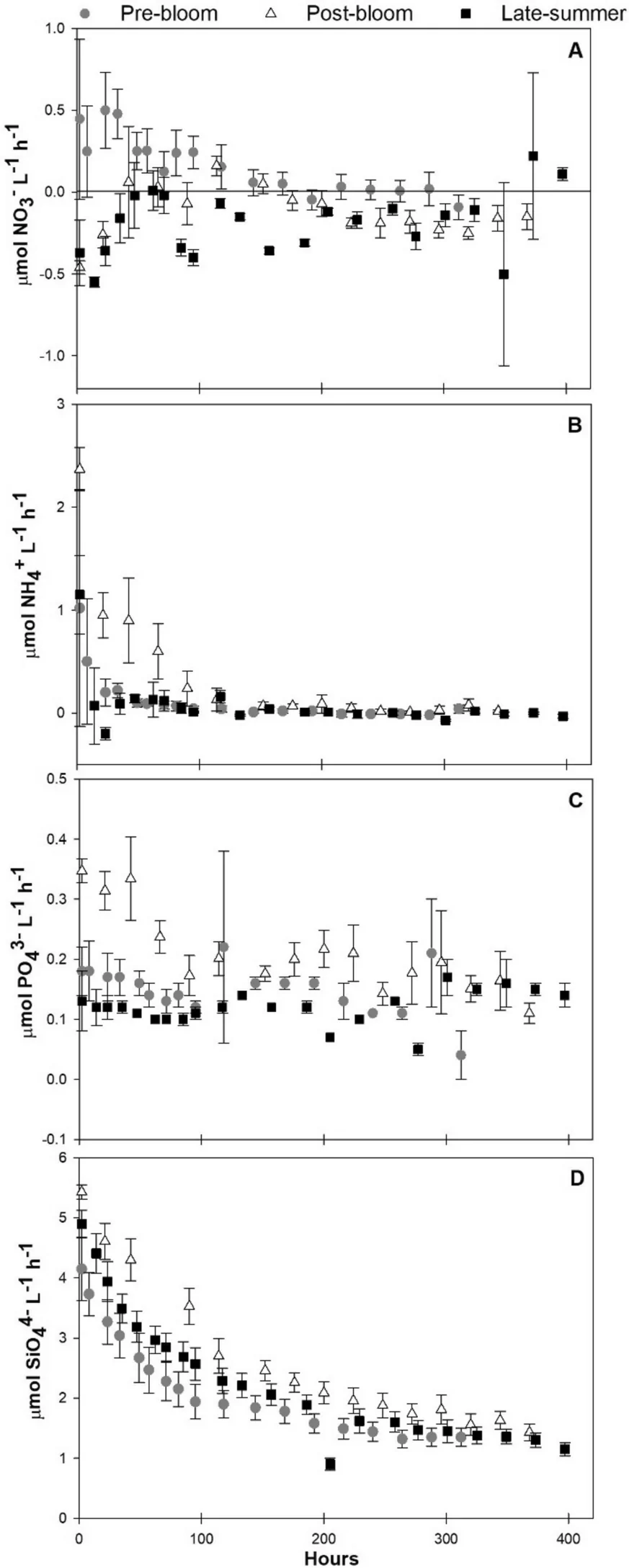
Seasonal rates (pre-bloom grey circles; post-bloom open triangles; late summer black squares) from FTR incubations of **A** NO_3_^−^ (μmol L^−1^ h^−1^) **B** NH_4_^+^ (μmol L^−1^ h^−1^) **C** PO_4_^3−^ (μmol L^−1^ h^−1^) and **D** SiO_4_ (μmol L^−1^ h^−1^). Error bars represent ± 1 standard deviation of the triplicate incubations

Calculated consumption or production of all solutes from the flow-through incubations showed initial variability in the signals measured during the first 2–3 days of the incubation period (Fig. 3 & 4). After this period, rates were stable and reveal some clear differences across the seasons (Table 2).

**Table 2:** Comparison of advective rates across seasons including oxygen consumption, nutrients and dissolved organics. (Data reported as mean ± 1 standard deviation, ranges in brackets)

O_2_ consumption μmol L^−1^ h^−1^	Fe(II) nmol L^−1^ h^−1^	DOC μmol L^−1^ h^−1^	DON μmol L^−1^ h^−1^	NO_3_^−^ μmol L^−1^ h^−1^	NH_4_^+^ μmol L^−1^ h^−1^	PO_4_^3−^ μmol L^−1^ h^−1^	DSi μmol L^−1^ h^−1^
*Pre-bloom*							
−9.60 ± 1.68 (−4.46 to −10.78)	−0.29 ± 0.64 (−10.78 to −4.46)	−1.83 ± 2.55^†^ (−5.40 to 1.33)	−0.05 ± 0.13^†^ (−0.21 to 0.23)	0.08 ± 0.11^†^ (−0.09 to 0.25)	0.03 ± 0.04* (−0.02 to 0.09)	0.15 ± 0.03 (0.11 to 0.22)	1.76 ± 0.8 (1.32 to 2.47)
*Post-bloom*							
−15.65 ± 0.99^†^ (−17.48 to −14.16)	3.93 ± 2.70^†^ (0.54 to 7.66)	0.59 ± 0.93 (−1.02 to 2.30)	0.27 ± 0.15^†^ (0.08 to 0.57)	−0.11 ± 0.12 (−0.25 to 0.16)	0.07 ± 0.07 (0.01 to 0.24)	0.18 ± 0.03 (0.11 to 0.22)	2.09 ± 0.59 (1.43 to 3.53)
*Late summer*							
−9.05 ± 0.68 (−10.70 to −7.99)	−0.17 ± 0.22 (−0.46 to 0.16)	1.16 ± 2.53 (−3.84 to 5.65)	0.06 ± 0.10^†^ (−0.11 to 0.23)	−0.16 ± 0.20 (−0.50 to 0.22)	−0.01 ± 0.03* −0.07 to 0.04	0.12 ± 0.04† (0.05 to 0.17)	1.53 ± 0.37 (0.90 to 2.21)

^†^ p > 0.05 across all seasons, *p > 0.05 to one other season

Oxygen consumption rates across all seasons were greatest in the initial 2–3 days and all reduced to more stable levels for the remainder of the incubation period (Fig. 3A). Average oxygen consumption rates (Table 2) taken from this more stable period were similar pre-bloom (−9.60 ± 1.68 μmol L^−1^ h^−1^) and late summer (−9.05 ± 0.68 μmol L^−1^ h^−1^), however, post-bloom oxygen consumption rates were found to be almost two-fold higher (−15.65 ± 0.99μmol L^−1^ h^−1^) and were significantly different (*p* < 0.05). Throughout all incubations and seasons, outflow from the flow-through reactors remained oxic. Oxygen consumption rates in the first 14 h were broadly comparable with whole core incubations conducted in parallel to our measurements (Hicks et al. 2017).

We found stark differences in the Fe(II) consumption/production rates between seasons. There was small net consumption pre-bloom (−0.29 ± 0.64 nmol L^−1^ h^−1^) and in the late summer (−0.17 ± 0.22 nmol L^−1^ h^−1^) (Fig. 3B), but substantial net production of Fe(II) post-bloom (3.93 ± 2.70 nmol L^−1^ h^−1^) (Table 2), which despite a high degree of variability was significantly different (*p* < 0.05) to the other seasons.

Dissolved organic carbon and nitrogen consumption/production rates were also variable between seasons. The most notable feature of DOC was consistent net consumption during the pre-bloom incubation (Fig. 3C) with average rates of −1.83 ± 2.55 μmol L^−1^ h^−1^ (Table 2) which was significantly different (*p* < 0.05) to post-bloom and late summer incubations. Post-bloom and in the late summer, there was net production of DOC from the permeable sediment. DOC production was greatest in the late summer (1.16 ± 2.53 μmol L^−1^ h^−1^), and significantly higher than other seasons (*p* < 0.05). The picture for the DON was not quite as clear. We observed slight DON consumption pre-bloom (−0.05 ± 0.13 μmol L^−1^ h^−1^) (Fig. 3D) that was significantly different (*p* < 0.05) to the other two seasons. DON was produced in both post-bloom and late summer incubations, with the greatest production rate found post-bloom (0.27 ± 0.15 μmol L^−1^ h^−1^) and only a small production rate in the late summer (0.06 ± 0.10 μmol L^−1^ h^−1^).

Inorganic nutrients were varied and reveal some distinct seasonal trends (Fig. 4; Fig. S1). NO_3_^−^ showed production rates pre-bloom up to 0.5 μmol L^−1^ h^−1^ for ~ 5 days before the system became balanced (Fig. 4A). However, during the post-bloom and late summer incubations a completely different scenario was observed, where the net consumption of NO_3_^−^ was measured and the largest consumption rate (−0.16 ± 0.20 μmol L^−1^ h^−1^) was during the late summer (Table 2). These latter incubations were significantly different (*p* < 0.05) to the pre-bloom rates of NO_3_^−^. NH_4_^+^ rates were unlike those obtained for NO_3_^−^, in that across all seasons there was an initial release and then all incubations reached a more balanced state (Fig. 4B). The greatest initial release was seen in the post-bloom incubation with rates of ~ 1 μmol L^−1^ h^−1^, which lasted for 4 days before reducing to only a marginal net release (0.07 ± 0.07 μmol L^−1^ h^−1^). During the late summer incubation, a marginal net consumption was measured (−0.01 ± 0.03 μmol L^−1^ h^−1^) which was significantly different (*p* < 0.05) to the post-bloom rates (Table 2). Net PO_4_^3−^ production was observed between all seasons, however temporal variability was found and the differences between seasons were significant (*p* < 0.05) (Fig. 4C). The greatest PO_4_^3−^ production rate occurred during the post bloom incubation (0.18 ± 0.03 μmol L^−1^ h^−1^) and the smallest rate was in the late summer (0.12 ± 0.04 μmol L^−1^ h^−1^) (Table 2). Dissolved silica (DSi) rates were interesting in that across all seasons there was a net release of DSi which decreased over the course of the incubation period (Fig. 4D). Differences between the seasons were found to be insignificant, however is should be noted that post-bloom rates were greater (2.09 ± 0.59 μmol L^−1^ h^−1^) than those pre-bloom (1.76 ± 0.8 μmol L^−1^ h^−1^) and in the late summer (1.53 ± 0.37 μmol L^−1^ h^−1^) (Table 2).

Incubations that were conducted to explore the potential implications of changing the residence time of fluid passing through the FTRs only revealed differences with regards to the oxygen consumption. The rates of the nutrients and Fe(II) (data not shown here) were similar both to the measurements obtained during the late summer field incubations as well as between the different residence times. However, the oxygen consumption rates across the range of residence time do reveal some variances (Table 3). The shorter the residence time the greater the consumption of oxygen (−16.02 ± 4.68 μmol L^−1^ h^−1^) in comparison to the incubation conducted with a longer residence time (−8.11 ± 1.02 μmol L^−1^ h^−1^). Differences between the longest and shortest residence time were found to be of significance (*p* < 0.05), with no significance found with the medium residence time.

**Table 3:** Oxygen consumption from FTR incubations with differing residence times. (Data reported as mean ± 1 standard deviation, ranges in brackets)

Column Length Residence time	O_2_ consumption μmol L^−1^ h^−1^
Short 8.62 h	−16.02 ± 4.68* (−27.29 to −10.32)
Medium 12.93 h	−12.39 ± 3.08 (−17.87 to −7.04)
Long 27.59 h	−8.11 ± 1.02* (−9.31 to −6.31)

^*^p > 0.05

## Discussion

Our data clearly demonstrate that the biogeochemical cycling of permeable sediments within the temperate Celtic Sea has seasonal variability. Similar seasonality was also shown by concurrent studies of respiration (Hicks et al. 2017), nitrogen cycling (Kitidis et al. 2017) and iron cycling (Klar et al. 2017). Kitidis et al. (2017), showed the surface increase in chlorophyll and concomitant decrease in CO_2_ partial pressure associated with the spring phytoplankton bloom in the Celtic Sea. The authors showed that this was followed by enhanced benthic respiration and annamox within 10 days and attributed this to transfer of organic matter to the seafloor. Upon examination of the physical characteristics of the sampling site, many of the differences observed particularly those for heterogeneity in grain size and percentage of fines fall within the variability found across the site. Whilst there was a significant increase in bulk density across the seasons, the high variability across the other sediment characteristics imply that the seasonal changes observed in the biogeochemistry of the solutes are controlled by factors other than the bulk sediment technical properties (Thompson et al. 2017).

### Controls on oxygen consumption

Given that the incubations remained oxic throughout the experimental run, we can be certain that the majority of the organic matter remineralisation was aerobic. Post-bloom oxygen consumption rates were almost double those measured pre-bloom and late summer. Oxygen dynamics are driven primarily by heterotrophic respiration, given the sample site depth of 100 m would eliminate the possibility of photosynthesis occurring. These enhanced oxygen consumption rates observed post-bloom coincided with an increase in organic carbon loading at the sampling site post-bloom. This is indicative of the delivery of phyto-detritus from the overlying water column, following the collapse of the pelagic spring bloom. Evidence and timing of the pelagic spring bloom can be observed in satellite imagery (MODIS; Thompson et al. 2017) which demonstrates the onset of the bloom in the second of week of April 2015. The bloom developed to its peak by the fourth week of April 2015 and then rapidly crashes by the second week of May 2015 and coincided with the sampling for the sediment incubations. Integrated POC concentrations in the overlying water column were 31% and 19% lower in winter and summer months respectively when compared with values from the spring bloom (Davis et al., 2019). Furthermore, measurements of POC and PON (integrated over water column depth) accumulation rates between 11th – 15th April were 61 mmol C m^−2^ d^−1^ and 10 mmol N m^−2^ d^−1^, respectively (Davis et al. 2019). Therefore, the increased oxygen consumption rates post-bloom highlights the heterotrophic response to the spring bloom organic material through enhancement of remineralisation. Oxygen penetration depths (OPD) post bloom were over three times shallower than those measured pre-bloom (this study; Hicks et al. 2017), further corroborating the seasonal change in oxygen dynamics.

We have established that the reduced oxygen consumption rates measured in the incubations pre-bloom and late summer are primarily driven by the lower carbon loads found in the permeable sediments, as the flow through the reactors was kept the same across seasons. However, when considering the sediment in situ, the current flow over the seabed needs to be addressed in order to fully understand the controls on the variable rates in oxygen consumption that are observed here. If we consider the OPD across the seasons, a 90% reduction is observed from pre-bloom to late summer. Given the reduction in organic carbon loading both within the water column and sediment, oxic remineralisation within the permeable sediment alone cannot explain the decrease in OPD. Despite the sampling site being at ~ 100 m depth, the currents over the bed are influenced daily by tidal processes with further variability seen in ADCP backscatter data across spring and neap tides (Thompson et al. 2019), with near-bed (30 cm above the bed) tidal currents reaching up to ~ 40 cm s^−1^ (Thompson et al. 2017). Additionally, wave orbital velocities at the sampling site are greatest pre-bloom (March 2015) and lowest in late summer (August 2015) with these wave effects influencing flow over the seabed (Thompson et al. 2019). The reduction in flow over the bed from pre-bloom to late summer would reduce both the speed and depth of the advective flow through the sediment and thus the delivery of solutes, including oxygen, organic carbon and nutrients (Janssen et al. 2005). This was evident in the shallower OPD observed in the late summer and further confirmed by the residence time incubations, which showed that an increase in fluid residence time and thus, a reduction in the delivery of solutes through the sediment significantly decreased the oxygen consumption rate.

Whilst regarding the role of organic carbon fuelling oxygen consumption it should be noted that pre-bloom there was a net drawdown of the DOC. This could imply that during this experiment, respiration of DOC may be fuelling the oxygen consumption, especially as POC was low in the sediment at this time and any POC that was present would likely be lacking in labile or semi-labile components after the low productivity observed in overlying surface waters throughout the winter months. Indeed, dissolved oxygen utilisation (DOU) measurements made from the same sampling site during the post-bloom cruise, indicate that microbial respiration was contributing to a greater degree than macrofaunal and meiofaunal respiration activities (Hick et al., 2017). Whilst there was no significant relationship found between oxygen consumption and DOC concentration, this could suggest a net drawdown of DOC from the water column driven by the microbial community during the winter months.

### Oxic release of iron(II)

Our data demonstrate a significant Fe(II) release during the post-bloom incubations following the breakdown of the pelagic spring bloom and delivery of phyto-detritus to the seafloor. The production of Fe(II) in marine sediment porewater is commonly attributed to microbial dissimilatory iron reduction (DIR), whereby Fe(III) oxides in the presence of organic matter (OM) are reduced to Fe(II) during the bacterial respiration of OM (Froelich et al. 1979). The release PO_4_^3−^ adsorbed to Fe oxide minerals may also be strongly coupled to this biological cycling of iron between Fe(III) and Fe(II) phases (Slomp et al 1998). Our results only show a concurrent release of Fe(II) and PO_4_^3−^ post-bloom, with no detectable Fe(II) production in the pre-bloom or late summer incubations. Given the sustained oxygenation in all our incubations, either DIR is restricted to small micro-environments or else it is not responsible for the production of Fe(II). Rather, it is the aerobic remineralisation of organic matter and release of inorganic solutes that supplies Fe(II) to porewater post-bloom.

In cohesive sediments with shallow OPD near to our study site in the Celtic Shelf Sea, diffusive fluxes of Fe(II) resulted from DIR and were also found to enter well-oxygenated bottom waters, where up to 70% of the dissolved Fe was present as Fe(II) (Klar et al. 2017). The study by Klar et al (2017) found that incubated Fe(II) oxidation rates were slower than predicted, which facilitated benthic exchange, and the greatest inhibition of Fe(II) oxidation rate was found in bottom water samples overlying sediment pore water, where the production of Fe(II)-binding organic ligands near the sediment–water interface was proposed as a mechanism to inhibit the oxidative loss of Fe(II) from solution. We suggest a similar inhibition of Fe(II) oxidation rates might account for our observations in permeable sediments of the same region. The main difference being that Fe(II) could be originating from the aerobic degradation of OM supplied in a discreet post-bloom period.

### Advective nutrient dynamics

N fluxes across the seasons varied with significant differences observed. Pre-bloom it is evidence that oxic remineralisation of organic matter resulted in the released of NH_4_^+^ which then underwent nitrification, evidenced by the decrease in NH_4_^+^ and release of NO_3_^−^. During this period a drawdown in DON was also observed. After this period a more balanced state was reached with negligible release of both NH_4_^+^ and NO_3_^−^. Post-bloom and late summer incubations reveal a different scenario where after an initial efflux of NH_4_^+^ (first 48 h) over the course of the incubations there was an overall net drawdown of NO_3_^−^. Whole core sediment–water flux incubations conversely reveal NO_3_^−^ efflux both pre-bloom and post-bloom (highest) and modest NO_3_^−^ drawdown in late summer (Kitidis et al. 2017). The differences in observations of NO_3_^−^ between the flow through reactor approach and intact core incubations could represent the limitations of the latter which do not capture advective processes adequately. The drawdown of NO_3_^−^ measured in the flow through reactors post-bloom and late summer suggests the presence of a NO_3_^–^ removal process. One such process, is denitrification (a strict anaerobic process) which may have taken place in micro-niche anaerobic habitats in what otherwise appear to be oxic permeable sediments (Santos et al. 2012). Indeed, observations made from SPI deployments revealed lenses of fine material within the permeable sediment (Fig. 2). Yet the sediment was homogenised prior to its introduction to the flow through reactors, so this is unlikely. Denitrification may have conceivably taken place in poorly ventilated recesses of the FTR, but Membrane Inlet Mass spectrometry (MIMS) analysis of outflow samples (N_2_:Ar ratio data not presented here) revealed no evidence of denitrification occurring during these incubations. Alternative NO_3_^−^ removal pathways such as annamox, hybrid denitrification, nitrifier denitrification, would also have resulted in substantial N_2_ formation (Spott et al., 2011). This was not observed in the flow through reactors employed here (as elevated N_2_:Ar) or the intact sediment core incubations using ^15^N tracers described by Kitidis et al. (2017). Whilst the processes of NO_3_^−^ removal identified in the FTR data are not clear, there is evidence that advective sediments may account for substantial N-loss. Over the last decade a number of studies including regions of the South Atlantic Bight (Rao et al. 2007), North Sea (Goa et al., 2010), and North-West Africa (Sokoll et al. 2016) have all demonstrated that microbes are capable of performing aerobic denitrification in advective permeable sediments. Here, bacteria simultaneously utilise both NO_3_^−^ and O_2_ as terminal electron acceptors in respiration (Robertson and Kuenen 1984). The study by Sokoll et al., (2016) which identified aerobic denitrification in permeable sediments on the North-West African shelf, found that the rates observed were four times higher than previously estimated from diffusive fluxes. Given the extent of permeable sediments in the global shelf seas, their role in oceanic N-loss is much greater than previously thought (Sokoll et al. 2016; Chua et al., 2022). Further study and consideration are required in temperate permeable shelf sediments to ascertain if indeed aerobic denitrification is a significant N-loss process here.

A release of PO_4_^3−^ to the overlying water column was witnessed across all three seasons with the greatest production rates measured post-bloom. Typically, in benthic environments, the release of PO_4_^3−^ from the sediment is strongly coupled with Fe(II) fluxes, as P is often adsorbed to Fe(oxyhydr)oxides under aerobic conditions, with release of both occurring in anaerobic, low pH environments via dissimilatory reductive dissolution of Fe (Slomp et al. 1998). The oxic incubation conditions across all seasons would therefore rule out the PO_4_^3−^ effluxes being the result of reduction of the Fe-oxides and this is further corroborated by the lack of Fe(II) release pre-bloom and in the late summer. However, since the post-bloom incubation had the greatest PO_4_^3−^ production in association with the only significant Fe(II) production, it appears that there could be some degree of DIR occurring in the micro-anaerobic environments as discussed previously. However, it is most likely given the oxic nature of permeable sediments, that the majority of the PO_4_^3−^ released across all seasons results from the aerobic remineralisation of organic matter where the resultant PO_4_^3−^ is not sequestered within the sediment due to saturation of the sorption sites and thus, released out into the overlying water column (Zhang and Huang 2007). This is evident with the greatest release of PO_4_^3−^ being witnessed post-bloom when organic matter was in most abundance following the crash of the pelagic spring bloom.

The fact that PO_4_^3−^ rates of production remained fairly consistent over the course of the incubations could represent a possible secondary contribution from the remineralisation of the dissolved organic P pool (DOP). As inflow waters were filtered prior to entering the incubation vessels, there was no replenishment of particulate organic matter (POM) to the sediments. Therefore, if the release of PO_4_^3−^ was solely down to remineralisation of particulate organic matter, a decrease in the fluxes would have been observed. DOP in the permeable sediment could be produced in-situ in the permeable sediment as a bi-product from metabolic activity (Payton and McLaughlin, 2007) or advected in from the overlying water column. Enzymatic assays have revealed that the hydrolytic enzyme alkaline phosphatase can be active in marine sediments (Kobari & Taga, 1979; Steenbergh et al. 2011) thus providing a pathway for the release of PO_4_^3−^ from DOP. Whilst this a plausible argument for the observations made during this study, further work is required to understand fully the role and dynamics of DOP cycling in permeable marine sediments.

Across all season’s rates of DSi production consistently declined over the course of the incubations, with no significant differences across seasons though post-bloom rates were marginally elevated. Had the incubations been run longer, they may have reached a scenario where the release of DSi could have continued to decrease until the system became balanced with no net release. This situation was not observed in any of the other solute rates and could be indicative of the nature of the Si cycle in marine systems.

DSi found in marine sediment pore waters can be the result of dissolution or remineralisation of biogenic silica (BSi), release from solids phases via coprecipitation with other metals (e.g. aluminium, iron, potassium etc.) or formation of authigenic minerals (Aller 2014).

The major source of the BSi to shelf sea sediments is from the delivery of diatom-derived marine detritus to the sediment interface from productive surface waters (Tréguar et al., 1995). This particulate material then undergoes dissolution at the sediment–water interface and releases DSi back into the water column (Frings et al., 2017). Within a permeable sediment, the BSi particles would be entrained into sediment pore waters via the advective flow, and this dissolution would occur within the sediment with the resultant DSi being fluxed back out to the overlying water column. As mentioned previously, the inflow waters were filtered thus there was no replenishment of BSi to the sediment and this could explain the consistent decrease in DSi rates over the incubation period. Correlation analysis of DSi with O_2_ consumption rates reveal significant correlations both pre-bloom (*r* = −0.86 *p* < 0.01) and late summer (*r* = −0.87 *p* < 0.01) which are indicative of microbially driven remineralisation of BSi (Holstein & Hensen, 2010) and represents an additional mechanism for DSi to be released from sediments. A significant correlation was not found post-bloom suggesting that remineralisation of BSi was not the key mechanism here, but rather dissolution. Indeed, it would be expected that the POM found in the permeable sediment post-bloom would be rich in BSi after the deposition of spring bloom detrital material, and would be readily dissolvable (Tréguar and De La Rocha, 2013) without the need for microbial action to release the DSi. However, it should be noted that during the spring bloom of 2015, the phytoplankton community was dominated by nanoplankton (2–20 μm) (Sharples et al., 2019) and not the larger celled diatoms that are typically known to drive the spring bloom in the North East Atlantic (Rees, et al. 1999). Therefore, the DSi rates measured in this study post-bloom could be lower than would typically be released from the dissolution of BSi-rich detritus in the region.

### Importance of permeable shelf sediments to the global ocean

We show the seasonal influence on rates of solute consumption and release in temperate permeable shelf sediments are driven primarily by the current flow over the bed and secondly by the supply of organic detrital material from the euphotic zone. O_2_ respiration rates align with previously measured rates in the North Sea (Ahmerkamp et al 2017, 2020) though do fall on the lower limits of these. This is expected given the Celtic Sea study site presented here is considerably deeper than that of the North Sea sites where wave and tidal influence is more evident resulting in a stronger advective flow through the permeable sediment. Furthermore, at shallower near-coastal sites the pelagic productivity is greater with reduced transport and remineralisation of particulate matter through the water column (Martin et al. 1987), resulting in more organic C being delivered to the sediments.

To ascertain the relative fluxes of solutes in the permeable sediments of the Celtic Sea we followed Ahmerkamp et al (2017) and integrated rates of consumption or production over the oxygen penetration depth and accounted for the porosity of the sediment. We found that oxygen fluxes across the sediment–water interface were greatest pre-bloom (−5.9 mmol O_2_ m^−2^ d^−1^) with fluxes decreasing post-bloom (−2.6 mmol O_2_ m^−2^ d^−1^) and late summer (− 0.4 mmol O_2_ m^−2^ d^−1^) which differs from the seasonal pattern in O_2_ consumption rates. This change in seasonal pattern is due to the OPD decreasing across the seasons and highlights the reduction in flow over the sea bed. Whilst again these O_2_ fluxes fall on the lower side of previously reported values (e.g. Ahmerkamp et al. 2017; Reimers & Fogaren 2021) consideration should be taken into the aerial extent of permeable sediments in the Celtic Sea. Here, 16% of the sea floor is found to be pure sandy sediments equating to an area of approximately 11,200 km^2^, which if we use to scale up the average O_2_ fluxes across the seasons finds ~ 3 × 10^4^ mol O_2_ d^−1^ are consumed in permeable sediments in the Celtic Sea. When regarding the role of this O_2_ consumption on the remineralisation of organic C in the permeable sediments of the Celtic Sea, we can apply a respiratory quotient (RQ, C_org_:O_2_) of 0.85 for outer shelf sediments from Jørgensen et al (2022) resulting in ~ 10 kmol C yr^−1^ being remineralised. Both the O_2_ fluxes and remineralisation of C calculated here should be taken as conservative estimates given that 64% of the Celtic Sea floor is covered in other permeable sediments (including gravelly sands) (Stephens 2015; Stephens & Diesing 2015; Thompson et al. 2017) which would also be subject to advective processes and contribute to O_2_ consumption in this shelf environment.

The Celtic Shelf Sea is seasonally stratified where nutrients are exhausted in the euphotic zone in late spring through to early autumn (Sharples et al., 2019) with convection, tides and elevated winds mixing the water column during winter (Ruiz-Castillo et al., 2019) replenishing nutrients to surface waters. The persistent release of both PO_4_^3−^ and DSi across all seasons from permeable sediments in the Celtic Shelf Sea could represent a significant source of these often-limiting nutrients to the overlying water column. Estimation of average fluxes across the seasons (using Amerkamp et al., (2017) flux calculation) were found to be 42 μmol PO_4_^3−^ m^2^ d^−1^ and 500 μmol DSi m^2^ d^−1^. PO_4_^3−^ fluxes are similar to those measured in the North Sea (De Borger et al. 2021) whilst DSi advective fluxes were considerably greater than those previously recorded for diffusive fluxes in the North East Atlantic (Tréguer and De La Rocha 2013). The fate of these nutrients in the bottom waters of the Celtic Sea is dependent on the mixing and transport of the water column. A proportion of the bottom waters in the Celtic Sea get transported off the shelf and into the North East Atlantic via Ekman drain, particularly during summer months (Holt et al. 2009; Graham et al. 2018), which would result in a loss of PO_4_^3−^ and DSi out of the region. However, given the complete collapse of the spring–summer stratification in the winter months and subsequent mixing of the water column (Ruiz-Castillo et al. 2019; Wihsgott et al. 2019), the PO_4_^3−^ and DSi supplied from permeable sediments could contribute to fuelling the spring bloom in the Celtic Sea.

We also revealed the release of Fe(II) post-bloom as result of aerobic remineralisation of OM and posit that its facilitated by organically stabilised Fe(II) present in the OM degradation products. Fe(II) supplied by aerobic degradation of OM may be a previously undescribed mechanism of Fe contribution from permeable sediments in the Celtic Shelf Sea and may provide an additive explanation to the apparent Fe(II) inhibition observed by Klar et al., (2017). Surface waters of the Celtic Shelf Sea become depleted during the summer months (Birchill et al., 2017), therefore the supply of Fe from the benthos could be a vital source helping to fuel the spring bloom. The flux of Fe(II) post-bloom (using Amerkamp et al. (2017) flux calculation) is 0.7 μmol Fe(II) m^2^ d^−1^ which equates to ~ 3% of fluxes estimated from diffusive sediments in the Celtic Sea (Klar et al. 2017). However, given the extent of sandy sediments across the Celtic Sea and when scaled up, Fe(II) fluxes amount to 7.5 mol Fe(II) d^−1^ post-bloom. Again, this is likely a conservative estimate given that the area of UK shelf seas covered with permeable sediments (~ 80%) is far greater than that covered by diffusive sediments (~ 20%) (Stephens 2015; Stephens & Diesing 2015; Thompson et al. 2017).

These findings from the Celtic Shelf Sea permeable sediments can be indicative of all temperate shelf seas globally, which are dominated by permeable sediments and experience the large variation in seasonal productivity and current flow. Furthermore, whilst over the last decade our knowledge and understanding of shelf sea environments has developed and the importance of permeable sediments in the biogeochemical cycling of nutrients and carbon is emerging, we need to turn our attention to the future. The North-Eastern Atlantic Shelf Sea, like other temperate shelf seas, has already warmed by 1 °C and is projected to rise to 2.9 °C by 2098 (Tinker et al. 2016) with the occurrence and intensity of marine heatwaves also increasing (Simon et al. 2023). This warming will impact the magnitude and length of the seasonal stratification of the water column leading to changes in nutrient supply and thus impact primary productivity and the flux of C to the seabed (Moore et al. 2018). Additionally, warming of the benthic environment will no doubt lead to changes in the metabolism, functioning and presence of organisms from microbes (Siedel et al., 2022) to larger fauna (Hiddink et al., 2015), thus influencing the remineralisation of organic matter and flux of C and nutrients to the overlying water column. Uncertainty remains as to how the processes and fluxes discussed in this present study will respond to a changing shelf sea.

## Conclusion

The biogeochemical cycling in permeable advective sediments of the Celtic Shelf Sea exhibit a seasonality that is driven by changes in the delivery of organic carbon from overlying pelagic waters and strength of current flow over the sea-bed. The findings from this study reveal for the first time that permeable sediments found in deeper shelf waters do contribute substantially to the remineralisation of organic matter, consumption of O_2_ and subsequent releases of essential nutrients and Fe. The delivery of OM from the overlying water column following the spring bloom, results in increased aerobic remineralisation, whilst during summer month the reduction in speed and strength of advective flow and subsequent pumping through the permeable sediment results in a decrease in the delivery of solutes and O_2_. The recycling function of permeable shelf sediments plays a key role in the supply of PO_4_^3−^, dSi and Fe(II) to the shelf waters and potential sustainment of primary production in the Celtic Sea, particularly in mid to outer shelf environments where fluvial inputs of nutrients and metals are only minor. The consequences of anthropogenic disturbance, changing climate and warming shelf seas will alter the function and capability of permeable sediments in the biogeochemical cycling of carbon and nutrients in the future. It is therefore imperative that we focus our research efforts into how these permeable shelf sediments respond to such pressures.

## Supplementary Information

Below is the link to the electronic supplementary material.

Supplementary file1 (DOCX 207 KB)

## Data Availability

The consumption/release rate data used for the visualisation and further analysis of the biogeochemical cycling of the permeable sediments in this study are available from the British Oceanographic Data Centre. Reynolds et al. 2026. https://doi.org/10.5285/5573c96a-9a4d-7714-e063-7086abc03e1c.
