# Supplementary material for: Seasonal biogeochemical cycling of permeable sediments in a temperate shelf sea environment
Source: Biogeochemistry. 2026 Aug 15;169(6):71. doi: 10.1007/s10533-026-01365-5 (PMC13751728; doi:10.1007/s10533-026-01365-5)
Supplement: Supplementary file 1 [file 10533_2026_1365_MOESM1_ESM.docx]

***Seasonal biogeochemical cycling of permeable sediments in a temperate shelf sea environment***

**Biogeochemistry**

S. E. Reynolds^1^, C.E.L. Thompson^2^, W.B. Homoky^3^, M. Williams^4^, J. Klar^2^, L.O. Amoudry^5^, V. Kitidis^6^, B. Silburn^7^, L. Chapman-Greig^1^, R. E. Parker^7^, M. Woodward^6^, P. J. Statham^2^, G. R. Fones^1^.

^1^School of the Environment & Life Sciences, University of Portsmouth, King Henry Building, King Henry I Street, Portsmouth, PO1 2DY, UK.

^2^School of Ocean and Earth Science, University of Southampton, National Oceanography Centre, European Way, Southampton, SO14 3ZH, UK.

^3^School of Earth and Environment, University of Leeds, Leeds, LS2 9JT, UK.

^4^Departamento de Ingeniería Hidráulica y Ambiental, Pontificia Universidad Católica de Chile, Santiago 7820436, Chile

^5^National Oceanography Centre, Joseph Proudman Building, 6 Brownlow Street, Liverpool, L3 5DA, UK.

^6^Plymouth Marine Laboratory, Prospect Place, Plymouth, PL1 3DH, UK

^7^Cefas, Pakefield Road, Lowestoft, Suffolk, NR33 0HT, UK

Corresponding author: Dr Sarah Reynolds ([sarah.reynolds@port.ac.uk](mailto:sarah.reynolds@port.ac.uk))

orcidID: 0000-0001-8700-0051

**Supplementary Information**

***Size characterisation of sediments***

The supplementary information included here provides additional insight to the properties of the permeable sediment that was sampled and utilised in the flow through incubations. The data includes in-depth size characterisation of the permeable sediment and inorganic nutrient depth profiles between 0 – 20 cm. Both sets of data reveal seasonal variations in these properties, further highlighting the dynamic nature of permeable sediments in temperate shelf seas.

**Table S1**: Sediment size characterisation for the three seasons.

|  | **Pre-bloom** | **Post-bloom** | **Late summer** |
| --- | --- | --- | --- |
| % GRAVEL | 0.6% | 0.3% | 0.6% |
| % SAND | 97.0% | 98.6% | 99.4% |
| % MUD | 2.4% | 1.2% | 0.0% |
| % FINE GRAVEL | 0.0% | 0.1% | 0.0% |
| % V FINE GRAVEL | 0.5% | 0.2% | 0.6% |
| % V COARSE SAND | 2.4% | 1.0% | 2.6% |
| % COARSE SAND | 18.3% | 8.8% | 23.5% |
| % MEDIUM SAND | 62.4% | 64.8% | 65.2% |
| % FINE SAND | 13.3% | 23.5% | 7.8% |
| % V FINE SAND | 0.6% | 0.5% | 0.3% |
| % V COARSE SILT | 0.4% | 0.3% | 0.0% |
| % COARSE SILT | 0.6% | 0.3% | 0.0% |
| % MEDIUM SILT | 0.5% | 0.2% | 0.0% |
| % FINE SILT | 0.4% | 0.2% | 0.0% |
| % V FINE SILT | 0.3% | 0.1% | 0.0% |
| % CLAY | 0.2% | 0.1% | 0.0% |

Detailed sediment size characterisation analysis reveals that the percentage of fine material present in the permeable sediment decreased across seasons from pre-bloom to later summer (Table S1).

***Estimation of porewater transport velocity.***

The pore water velocity is commonly related to a pressure gradient

$$\begin{aligned} q=\alpha\frac{\kappa}{\nu}\frac{1}{L}\frac{\Delta P}{\rho} \#\left( 1 \right) \end{aligned}$$

where $q$ is the pore water transport velocity, $\kappa$ the sediment permeability (in m^2^), $\nu$ the kinematic viscosity of water, $\rho$ the density of water, $\Delta P$ the pressure difference inducing the pore water flow, $L$ a length scale. $\alpha$ is a constant the value of which varies in the literature: i.e. $\alpha=1$ (Shum, 1993, 1992) or $\alpha=2\pi$ (Elliott and Brooks, 1997; Rutherford et al., 1995). We follow different approaches to estimate the pressure gradient for currents over ripples or for waves over ripples.

For waves over ripples, we follow the approach by Shum (1993, 1992) who related the pressure gradient over rippled beds under surface progressive gravity waves to the bottom orbital velocity.

$$\begin{aligned} \frac{\Delta P}{\rho}=u_{b}^{2}\frac{\pi s}{\left( 1-\pi s \right)^{2}\left( 1+\pi s \right)^{2}}\#\left( 2 \right) \end{aligned}$$

where $u_{b}$ is the amplitude of the bottom orbital velocity, and $s=\eta/\lambda$ is the ripple steepness with $\eta$ the ripple height and $\lambda$ the ripple length. This results in the wave-driven porewater velocity, $q_{w}$, to be $\begin{aligned} q_{w}=\frac{\kappa}{\nu}\frac{u_{b}^{2}}{\lambda}\frac{\pi s}{\left( 1-\pi s \right)^{2}\left( 1+\pi s \right)^{2}}\#\left( 3 \right) \end{aligned}$

In turn, the bottom orbital velocity can be estimated from surface wave characteristics. For monochromatic waves, linear wave theory gives

$$\begin{aligned} u_{b}=\frac{H\pi}{Tsinh\left( kh \right)} \#\left( 4 \right) \end{aligned}$$

where $H$ is the wave height, $T$ is the wave period, $h$ is the water depth, and $k$ is the wavenumber. However, note that applying the formula above directly to surface wave spectra will be less accurate than estimates made from the full spectra, as outlined in Wiberg and Sherwood (2008).

Taking a wave height of 10 m, which corresponds to a 50-year return period for the region (Bricheno et al., 2015), and a period of 15 s as an indicative worst-case scenario for $q_{w}$, and using the permeability values of table 1, we obtain values in the range of 1-2 cm h^-1^.

For pressure gradients resulting from the interaction between currents and bedforms, the model of Elliott and Brooks (1997) uses an empirical expression following which the pressure gradient depends on the mean water depth. Such an expression would not be valid for marine bedforms in coastal and shelf seas. Instead, we follow the approach of Musa et al. (2014), which provides the pressure over a rippled bed in a planetary boundary layer

$$\begin{aligned} \frac{\Delta P}{\rho}\approx su_{c}^{2}\#\left( 5 \right) \end{aligned}$$

where $u_{c}$ is the near bed current velocity. This leads to

$$\begin{aligned} q_{c}=\frac{\kappa}{\nu}\frac{u_{c}^{2}}{\lambda}s\#\left( 6 \right) \end{aligned}$$

Observed near-bed current reached approximately 40 cm s^-1^, and ripple steepness was observed in situ to be $s\approx0.1$. Using the permeability values of table 1, we obtain values in the range of 0.1 to 0.2 cm h^-1^.

***Inflow and outflow data***

Inflow measurements of the various solutes (Table S2) reveal that across all parameters some variability exists despite the inflow water being pre-filtered (0.2 μm) and kept under stable conditions. When calculating the advective rates the inflow solute measurement closest in time to the outflow measurement was used in the calculation (eq. 5 – main text).

Outflow measurements of oxygen reveal that across all seasons the FTRs remined oxic over the course of the incubation though there are clear seasonal differences with the post-bloom O_2_ concentration measuring on average at 71.98 μmol L^-1^.

**Table S2:** Inflow and outflow data from the flow through reactors. Outflow data represents the data collected after a stabilisation period – typically after 5 days, that has been used to calculate subsequent average consumption/production rates and fluxes.

| **INFLOW** |  | **Oxygen** | **Nitrate** | **Ammonium** | **Silicate** | **Phosphate** | **Iron(II)** |
| --- | --- | --- | --- | --- | --- | --- | --- |
|  |  | **µM** | **µM** | **µM** | **µM** | **µM** | **nM** |
| ***Pre-bloom*** | **average** | 270 | 1.92 | 0.18 | 3.61 | 0.32 | 13.72 |
|  | **std dev** | 24.19 | 0.56 | 0.22 | 0.09 | 0.24 | 1.95 |
|  | **n** | 4 | 2 | 2 | 2 | 2 | 2 |
|  |  |  |  |  |  |  |  |
| ***Post-bloom*** | **average** | 278 | 5.63 | 0.31 | 4.63 | 0.55 | 4.94 |
|  | **std dev** | 8.63 | 1.70 | 0.27 | 0.22 | 0.22 | 3.54 |
|  | **n** | 6 | 5 | 5 | 5 | 5 | 5 |
|  |  |  |  |  |  |  |  |
| ***Late summer*** | **average** | 272 | 4.55 | 1.20 | 5.03 | 0.47 | 7.59 |
|  | **std dev** | 7.68 | 2.33 | 1.33 | 0.57 | 0.34 | 6.53 |
|  | **n** | 18 | 18 | 18 | 18 | 18 | 8 |
| **OUTFLOW** |  |  |  |  |  |  |  |
| ***Pre-bloom*** | **average** | 131.95 | 2.88 | 0.50 | 26.36 | 2.31 | 9.96 |
|  | **std dev** | 30.14 | 1.59 | 0.46 | 5.24 | 0.73 | 10.94 |
|  | **n** | 13 | 13 | 13 | 13 | 12 | 13 |
|  |  |  |  |  |  |  |  |
| ***Post-bloom*** | **average** | 71.96 | 2.69 | 1.06 | 31.52 | 2.76 | 53.02 |
|  | **std dev** | 27.95 | 1.84 | 1.19 | 7.56 | 0.53 | 44.55 |
|  | **n** | 13 | 13 | 12 | 13 | 13 | 13 |
|  |  |  |  |  |  |  |  |
| ***Late summer*** | **average** | 156.28 | 1.53 | 0.73 | 24.96 | 1.97 | 6.18 |
|  | **std dev** | 27.63 | 0.76 | 0.55 | 5.02 | 0.38 | 4.76 |
|  | **n** | 12 | 12 | 12 | 12 | 12 | 12.00 |

***Nutrient profiles***

**Figure S1** Seasonal nutrient (μmol L^-1^) pore water profiles of (a) NO_3_^-^ (b) NH_4_^+^ (c) PO_4_^3-^ and (d) SiO_4_^4-^.

Inorganic nutrient profiles (Fig. S1) demonstrate temporal variability across all four (NO_3_^-^, NH_4_^+^, PO_4_^3-^ and SiO_4_^4-^) parameters measured. Differences in the NO_3_^-^ profiles were found not to be significant, however, some differences are worth noting. Across all seasons, NO_3_^-^ concentrations exhibited a maximum at depth below the sediment-water boundary (Fig. S1A) with the depth of this maxima being greater and broader pre-bloom (3-7 cm, 7.93-9.48 μM NO_3_^-^) in comparison to post-bloom (3 cm, 12.89 μM NO_3_^-^) and late-summer (2 cm, 8.57 μM NO_3_^-^) following the same trend as witnessed in the OPD. Succeeding these maxima, concentrations of NO_3_^-^ decreased with depth to 2-3 μM NO_3_^-^. NH_4_^+^ concentrations exhibit a similar profile across all seasons, with low concentrations at the surface which increases with depth (Fig. S1B). At the sediment surface concentrations ranged from 0.12 μM NH_4_^+^ pre-bloom, 0.47 μM NH_4_^+^ post-bloom and 1.32 μM NH_4_^+^ late summer. Substantial increases with depth were observed with the highest concentration found late summer (17 cm, 52.65 μM NH_4_^+^) and the lowest concentration pre-bloom (17 cm, 40.91 μM NH_4_^+^). Differences between the seasons for NH_4_^+^ were found to be not significant. PO_4_^3-^ concentrations were also found to be low at the sediment water boundary, with concentrations increasing across seasons from 0.23 μM PO_4_^3-^ pre-bloom to 0.64 μM PO_4_^3-^ late summer (Fig. S1C). Profiles increased with depth to a maxima at 10 cm with 4.05 μM PO_4_^3-^ pre-bloom increasing to 5.78 and 5.71 μM PO_4_^3-^ post-bloom and late summer respectively. Concentrations then decreased to ~2.5 μM PO_4_^3-^ pre-bloom and late summer with only a slight decrease observed post-bloom (4.88 μM PO_4_^3-^). Differences in PO_4_^3-^ were found to be of significant difference (*p* <0.05) between pre-bloom and post-bloom. Profiles for SiO_4_^4-^ μM also follow a pattern of depleted concentrations in the surface sediment and increasing with depth (Fig. S1D). Significant differences (*p* <0.001) were found between pre-bloom and post-bloom and pre-bloom and late summer. Concentrations of SiO_4_^4-^ at the sediment-water interface were 3.15 μM SiO_4_^4-^ pre-bloom with a substantial increase to 17.73 μM SiO_4_^4-^ post-bloom, decreasing back to 3.92 μM SiO_4_^4-^ late summer. The largest increase with depth occurs post-bloom and late summer with concentrations reaching 103 μM SiO_4_^4-^ and 108 μM SiO_4_^4-^ respectively. Pre-bloom concentrations reach a maxima of 84.81 μM SiO_4_^4-^ at 17 cm.

**References:**

Bricheno, L.M., Wolf, J., Aldridge, J., 2015. Distribution of natural disturbance due to wave and tidal bed currents around the UK. Cont. Shelf Res. 109, 67–77. https://doi.org/10.1016/j.csr.2015.09.013

Elliott, A.H., Brooks, N.H., 1997. Transfer of nonsorbing solutes to a streambed with bed forms: Theory. Water Resour. Res. 33, 123–136. https://doi.org/10.1029/96WR02784

Musa, R.A., Takarrouht, S., Louge, M.Y., Xu, J., Berberich, M.E., 2014. Pore pressure in a wind-swept rippled bed below the suspension threshold. J. Geophys. Res. Earth Surf. 119, 2574–2590. https://doi.org/10.1002/2014JF003293

Rutherford, J.C., Boyle, J.D., Elliott, A.H., Hatherell, T.V.J., Chiu, T.W., 1995. Modeling Benthic Oxygen Uptake by Pumping. J. Environ. Eng. 121, 84–95. https://doi.org/10.1061/(ASCE)0733-9372(1995)121:1(84)

Shum, K.T., 1993. The effects of wave-induced pore water circulation on the transport of reactive solutes below a rippled sediment bed. J. Geophys. Res. Oceans 98, 10289–10301. https://doi.org/10.1029/93JC00787

Shum, K.T., 1992. Wave-induced advective transport below a rippled water-sediment interface. J. Geophys. Res. Oceans 97, 789–808. https://doi.org/10.1029/91JC02101

Wiberg, P.L., Sherwood, C.R., 2008. Calculating wave-generated bottom orbital velocities from surface-wave parameters. Comput. Geosci., Predictive Modeling in Sediment Transport and Stratigraphy 34, 1243–1262. https://doi.org/10.1016/j.cageo.2008.02.010
